# Assessment of ventricular electrical heterogeneity in left bundle branch pacing and left ventricular septal pacing by using various electrophysiological methods

**DOI:** 10.1111/jce.16435

**Published:** 2024-09-23

**Authors:** Jesse H. J. Rijks, Luuk Heckman, Sjoerd Westra, Richard Cornelussen, Subham Ghosh, Karol Curila, Radovan Smisek, Domenico Grieco, Edoardo Bressi, Uyên Châu Nguyên, Joost Lumens, Antonius M.W. van Stipdonk, Dominik Linz, Frits W. Prinzen, Justin G. L. M. Luermans, Kevin Vernooy

**Affiliations:** ^1^ Department of Cardiology, Cardiovascular Research Institute Maastricht (CARIM) Maastricht University Medical Center+ (MUMC+) Maastricht The Netherlands; ^2^ Department of Physiology, Cardiovascular Research Institute Maastricht (CARIM) Maastricht University Maastricht The Netherlands; ^3^ Department of Cardiology Radboud University Medical Center (RadboudUMC) Nijmegen The Netherlands; ^4^ Bakken Research Center Maastricht The Netherlands; ^5^ Medtronic Fridley Minnesota United States of America; ^6^ Department of Cardiology, Third Faculty of Medicine Charles University and University Hospital Kralovske Vinohrady Pregue Czechia; ^7^ The Czech Academy of Sciences, Institute of Scientific Instruments Brno Czechia; ^8^ Department of Cardiology Policlinico Casilino of Rome Rome Italy; ^9^ Department of Biomedical Engineering, Cardiovascular Research Institute Maastricht (CARIM) Maastricht University Maastricht The Netherlands; ^10^ Centre for Heart Rhythm Disorders, Royal Adelaide Hospital The University of Adelaide Adelaide South Australia Australia; ^11^ Department of Biomedical Sciences, Faculty of Health and Medical Sciences University of Copenhagen Denmark

**Keywords:** bradycardia pacing, cardiac resynchronization therapy, conduction system pacing, dyssynchrony, left bundle branch area pacing

## Abstract

**Introduction:**

Left bundle branch area pacing (LBBAP) comprises pacing at the left ventricular septum (LVSP) or left bundle branch (LBBP). The aim of the present study was to investigate the differences in ventricular electrical heterogeneity between LVSP, LBBP, right ventricular pacing (RVP) and intrinsic conduction with different dyssynchrony measures using the ECG, vectorcardiograpy, ECG belt, and Ultrahigh frequency (UHF‐)ECG.

**Methods:**

Thirty‐seven patients with a pacemaker indication for bradycardia or cardiac resynchronization therapy underwent LBBAP implantation. ECG, vectorcardiogram, ECG belt and UHF‐ECG signals were recorded during RVP, LVSP and LBBP, and intrinsic activation. QRS duration (QRSd) was measured from the ECG, QRS area was calculated from the vectorcardiogram, LV activation time (LVAT) and standard deviation of activation time (SDAT) from ECG belt and electrical dyssynchrony (e‐DYS16) from UHF‐ECG.

**Results:**

Both LVSP and LBBP significantly reduced ventricular electrical heterogeneity as compared to underlying LBBB and RV pacing in terms of QRS area (*p* < .001), SDAT (*p* < .001), LVAT (*p* < .001) and e‐DYS16 (*p* < .001). QRSd was only reduced as compared to RV pacing(*p* < .001). QRS area was similar during LBBP and normal intrinsic conduction, e‐DYS16 was similar during LVSP and normal intrinsic conduction, whereas SDAT was similar for LVSP, LBBP and normal intrinsic conduction. For all these variables there was no significant difference between LVSP and LBBP.

**Conclusion:**

Both LVSP and LBBP resulted in a more synchronous LV activation than LBBB and RVP. Especially LBBP resulted in levels of LV synchrony comparable to normal intrinsic conduction.

## Graphical abstract

LBBAP = left bundle branch area pacing; LVSP = left ventricular septal pacing; LBBP = left bundle branch pacing; LBBB = left bundle branch block; RV = right ventricle; aLBBAP = LBBAP with anodal stimulation.

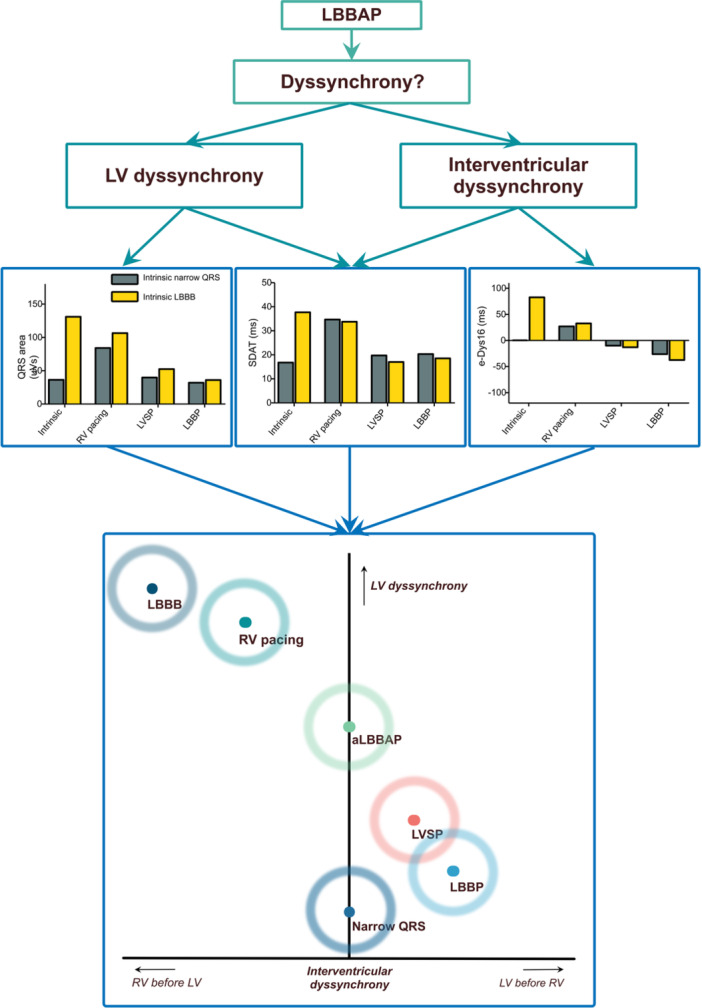

## 1 INTRODUCTION

Left bundle branch area pacing (LBBAP) with either left ventricular septal pacing (LVSP) or left bundle branch pacing (LBBP) is used as a more physiologic alternative to right ventricular pacing (RVP) to prevent pacing‐induced ventricular dyssynchrony and as an alternative to biventricular pacing (BiV‐CRT) in cardiac resynchronization therapy (CRT).^1^, ^2^, ^3^, ^4^, ^5^ In LVSP, there is capture of the myocardium at the left side of the interventricular septum (IVS), whereas in LBBP capture of the left bundle branch (LBB) is achieved.^6^ The MELOS study showed that LBB capture is achieved in almost 80 percent of LBBAP procedures in experienced hands.^7^ This leaves a significant proportion of patients undergoing LBBAP being treated with LVSP.

Both LVSP and LBBP are reported to result in a (near)‐physiologic electrical and mechanical activation of the left ventricle (LV).^3^, ^8^, ^9^, ^10^ Curila et al. showed that LBBP, when compared to LVSP, results in the more physiologic activation of the LV,^8^ but at the cost of a larger interventricular dyssynchrony.^8^ LVSP also results in (near)‐physiologic activation of the left ventricle, although the LV free wall is slightly delayed when compared to LBBP.^8^ However, the exact effect of additional LBB capture in terms of electrical dyssynchrony is not yet fully understood and was never studied using a combination of different methods of noninvasive ventricular dyssynchrony assessment.

The aim of the present study is to explore the ventricular electrical activation induced by LBBAP by comparing LBBP and LVSP in relation to RVP and intrinsic activation. A comprehensive array of diagnostic tools will be used, including conventional 12‐lead ECG, vectorcardiography, body surface potential mapping (ECG BELT), and ultra‐high frequency ECG (UHF‐ECG). Furthermore, this study aims to compare these methodologies to gain a deeper understanding of what the different measurements represent.

## 2 METHODS

### 2.1 Study design and patient selection

This study was designed as a multicenter, prospective, interventional cohort study performed in the Maastricht University Medical Center, Maastricht (MUMC+), and the Radboud University Medical Center (Radboudumc), Nijmegen. Patients referred for permanent pacemaker implantation for either bradycardia or cardiac resynchronization therapy (CRT) between may 2021 and December 2022 were included and underwent LBBAP. All patients provided written informed consent. The study was approved by the local ethics committee (METC 20‐099), the Dutch Central Committee on Research Involving Human Subjects (CCMO) and the institutional review boards of both participating centers. The study complied with the declaration of Helsinki.

**Figure 1:**
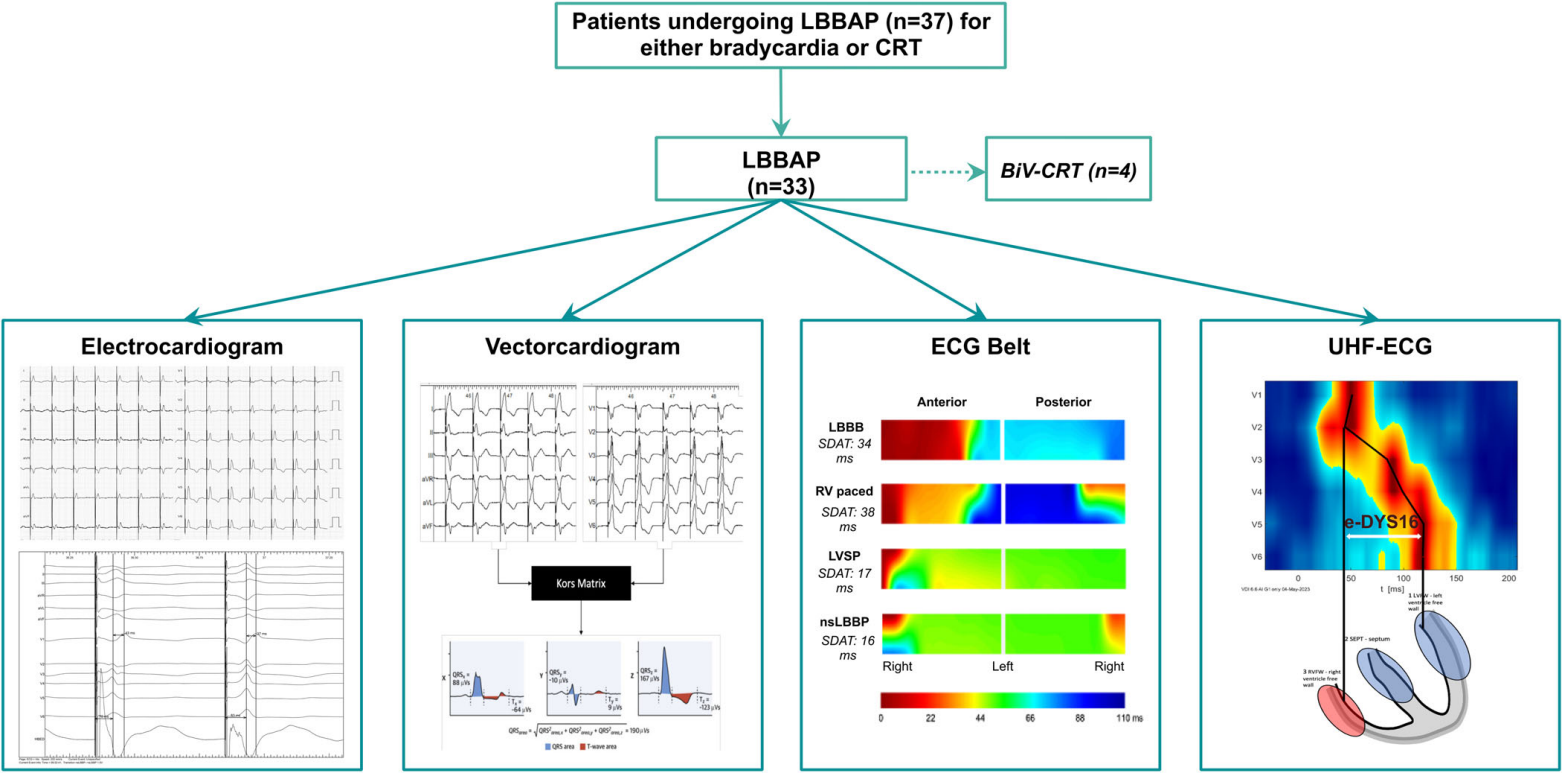
Study design and ventricular electrical heterogeneity assessment methodology. CRT, cardiac resynchronization therapy; LBBAP, left bundle branch area pacing; BiV‐CRT, biventricular cardiac resynchronization therapy; ECG, electrocardiogram; UHF‐ECG, ultraihigh frequency electrocardiogram.

The study design is presented in Figure 1.

### 2.2 Implantation procedure

All patients underwent LBBAP implantation with the Select Secure lead (model 3830, Medtronic, Minneapolis, MN, USA). The implantation was performed as described previously.^11^ In short, the location of the bundle of His was mapped as a reference point, either by recording an intracardiac electrogram from the tip of the LBBAP lead or by contrast angiogram of the tricuspid valve region. Thereafter, the lead was advanced ~1.5–2 cm towards the right ventricular apex. Pacemapping at the right side of the IVS was used to confirm the region of interest for lead penetration through the IVS, preferably resulting in a paced LBBB morphology with a negative, notched QRS in lead V1, a positive QRS in lead I and II and a negative QRS in lead III. Subsequently, the lead was screwed to the left side of the IVS.

LBBP was defined as: 1) an output‐dependent transition in QRS morphology, either from nonselective to selective LBBP or from nsLBBP to LVSP at decremental voltage output pacing; 2) interval from the left‐sided Purkinje potential to V6RWPT (time to peak R wave in lead V6) in intrinsic rhythm equal to pacing stimulus to V6RWPT ( ± 10 ms); 3) short and stable pacing stimulus to V6RWPT < 75 ms in narrow baseline QRS and <80 ms in LBBB/interventricular conduction delay; 4) V6–V1 interpeak time >44 ms.^11^ LVSP was defined as either a paced qR/Qr complex in lead V1 without evidence of LBBP or an output dependent transition from nsLBBP to LVSP with decreasing output according to the recent consensus on conduction system pacing.^11^ When neither evidence of LBBP nor evidence of LVSP was present as mentioned above, it was considered to be a failed LBBAP implantation.

In case of failure of LBBAP implantation, patients were treated as indicated according to the ESC guidelines on cardiac pacing and CRT.^12^ I.e. bradypacing patients (either sick sinus syndrome, AV block or pace & ablate) with LVEF ≥ 40% were treated with conventional RVP and bradypacing patients with LVEF < 40% and CRT patients (Class I or IIa indication for cardiac resynchronization therapy) were treated with conventional BiV‐CRT in case of LBBAP failure.

### 2.3 Pacing protocol

All patients underwent a pre‐specified pacing protocol with pacing performed at 10 bpm above intrinsic heart rate or at 80bpm in case of AV block (AVB). Pacing was performed in DDD mode with a programmed AV‐delay 60 ms shorter than intrinsic AV delay observed during AAI pacing to avoid fusion with intrinsic conduction or a programmed AV delay of 120 ms in case of AVB. In patients in atrial fibrillation during the implantation procedure, VVI mode was used.

Pacing was performed in different configurations: 1) AAI pacing with intrinsic conduction; 2) RVP; 3) unipolar LBBAP with high output (8 V@0.4 ms); 4) unipolar LBBAP with medium output (3 V@0.4 ms); 5) unipolar LBBAP at low output (0.1 V above threshold; 6) bipolar LBBAP with anodal stimulation (aLBBAP) if present. Pacing configuration two (RVP) was performed by placing a temporary pacing catheter at the RV apex. Pacing configuration three to six were performed at the final LBBAP pacing site.

In all LBBAP settings the capture type (either LVSP or ns/sLBBP) was determined using the criteria as mentioned above. Anodal stimulation was assessed by bipolar decremental output pacing. During bipolar decremental output pacing a QRS morphology change from a QS or Qr pattern to an rSr’ or QR/qR pattern in lead V1 can be found present.^13^ The output at which this morphology change occurred was defined as the anodal threshold. Anodal stimulation was defined as pacing above this anodal threshold.

### 2.4 Electrophysiological recording system

The 12‐lead ECG and local EGM from the tip of the pacing lead were recorded at a 200 mm/s sweep speed at all pacing sites using an EP Recording System (LABSYSTEM Pro, Boston Scientific, Marlborough, MA, USA, or Workmate Claris, Abbott, Plymouth, MN, USA) recording frequencies up to 500 Hz (Figure 1). Intrinsic QRS duration was measured from onset of QRS to end of QRS. QRS duration during pacing was measured from pacing stimulus to end of QRS. Pacing stimulus to V6RWPT was measured as the interval between the pacing stimulus and peak R‐wave in V6. Time to peak R‐wave in V1 (V1RWPT) was measured from pacing stimulus to r'/R' in V1. The interval between V6RWPT and V1RWPT was defined as V6‐V1 interpeak time according to Jastrzebski et al.^14^ Both V6RWPT and V6‐V1 interpeak time were used to confirm LBB capture as mentioned before.

### 2.5 Vectorcardiogram

The vectorcardiogram (VCG) is a method of recording the magnitude and direction of the electrical forces that are generated by the heart in a 3‐dimensional model. QRS area was calculated from the VCG at all pacing sites. The 2‐dimensional ECG was converted into a 3‐dimensional VCG. The VCG was constructed as described previously,^15^, ^16^ via the Kors conversion matrix ^17^ using custom MATLAB software (Mathworks Inc, Natick, MA, USA). QRS area was calculated as the sum of the area under the QRS complex in the calculated vectorcardiographic orthogonal X, Y, and Z lead (QRS area = (QRS_area,x_^2^ + QRS_area,y_^2^ + QRS_area,z_^2^)^1/2^) (Figure 1 *).* Mafi Rad et al. showed that QRS area can identify delayed left ventricular wall activation.^18^ Moreover, QRS area identifies delayed left ventricular lateral wall activation better than QRS duration and LBBB morphology.^18^ Therefore, as a good marker of left ventricular dyssynchrony, it was validated in CRT patients.^19^, ^20^

### 2.6 ECG belt

The ECG Belt System (Medtronic, Minneapolis, MN, USA) is a body surface mapping system consisting of a 2‐piece electrode array that wraps around the upper torso, containing approximately 20 electrodes on each array as well as four electrodes around the lower torso. The ECG Belt System provides beat by beat isochronal maps of the anterior and posterior torso and can quantify electrical dyssynchrony (Figure 1 *).* Standard deviation of activation times (SDAT) is calculated as a metric of global ventricular electrical heterogeneity^21^ (Figure 1 *).* Additionally, left ventricular activation time (LVAT) was calculated as a metric for the LV activation times from the left thoracic electrodes, with higher values of LVAT corresponding with LV dyssynchrony.

### 2.7 Ultra‐high frequency ECG

UHF‐ECG is a noninvasive method to display the sequence of ventricular activation and its resulting ventricular electrical (dys)synchrony (Figure 1).^22^ It has been used to describe differences in ventricular activation patterns between various types of pacing (RVP, LBBAP, BiV‐CRT).^8^, ^23^, ^24^

**Figure 2:**
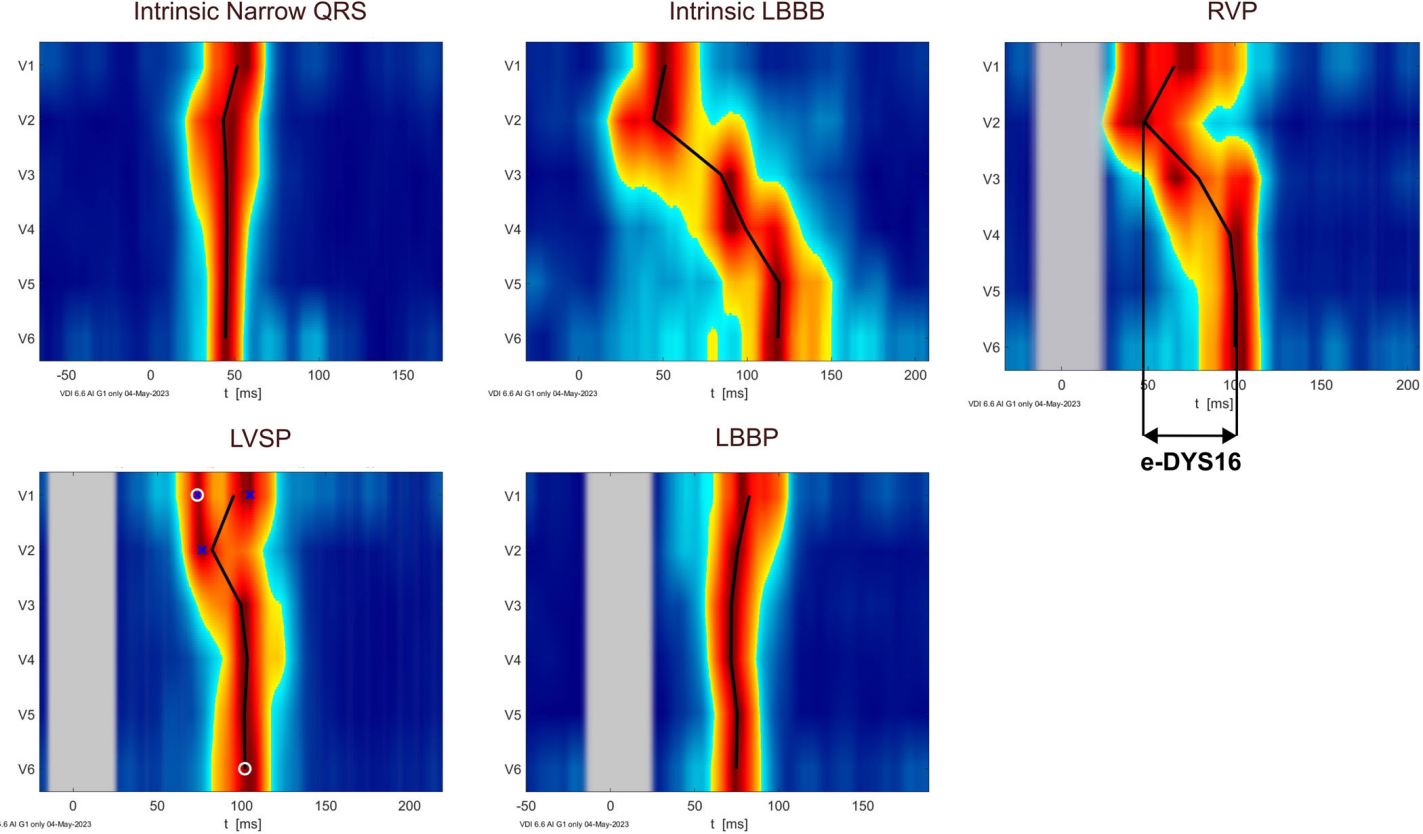
UHF‐ECG color maps. LBBB, left bundle branch block; RVP, right ventricular pacing; LVSP, left ventricular septal pacing; LBBP, left bundle branch pacing.

The 12‐lead ECG was recorded on the EP Recording System with a sampling rate of 4 kHz and frequencies up to 500 Hz. Amplitude frequency envelopes for six different frequency bands from 150 to 500 Hz (bandwith 100 Hz and steps of 50 Hz) are computed using the Hilbert transformation and are normalized for V1–V6 precordial leads. The signal from each precordial lead was displayed as a color map (Figure 1 and 2). Local activation times were calculated as the centre of mass of UHF‐QRS's above 50% threshold of the baseline‐to‐peak amplitude in each chest lead. e‐DYS16 was calculated as the maximal difference between the first and last activation in the precordial leads V1–V6 and represents interventricular dyssynchrony.^22^ UHF‐ECG was analyzed using VDI scientific software (VDI Technologies Inc., USA, 2023).

### 2.8 Data collection and analysis

Demographic data and medical history were collected at enrollment. Procedure related characteristics including procedure success, acute complications, procedural ECG and local EGM measurements and ECG Belt recordings at all pacing positions were collected during implantation. Post‐procedural VCG analysis was performed on ECG recordings extracted from the EP recording system with ectopic beats excluded. UHF‐ECG analysis was performed on 60 s recordings extracted from the EP recording system and were processed as described previously.^22^

### 2.9 Statistical analysis

Statistical analyses were performed using IBM SPSS statistics software version 29 (SPSS Inc., Chicago, IL, USA). Continuous variables are presented as mean ± SD or as median [interquartile range] according to distribution. Discrete variables are presented as count and proportion (%). Paired comparisons between two normally distributed variables were made using the paired student's *T*‐test. Repeated measurement comparisons and pairwise comparisons between different pacing positions were made using a linear mixed effects model. The results of these models are presented as estimated marginal mean [95% CI] and comparisons as mean differences [95% CI]. A *p*‐value < .05 was considered statistically significant.

## 3 RESULTS

### 3.1 Baseline characteristics

A total of 37 patients were included in this study. Patient characteristics are summarized in Table 1. The cohort was predominantly male (65%). The indications for implantation were pace‐and‐ablate in nineteen patients (51%), CRT in seven patients (19%), AV block in seven patients (19%) and sick sinus syndrome in four patients (11%). One‐third of patients had an LBBB on baseline ECG. Mean baseline QRS duration was 117 ± 35 ms, with a QRS duration of 98 ± 20 ms for patients with a narrow baseline QRS morphology (including RBBB QRS morphology) (nQRS) and a QRS duration of 159 ± 24 ms for patients with a baseline LBBB QRS morphology. LVEF at baseline was 46 ± 11%.

**Table 1:** Baseline characteristics.

	*N* = 37
Age, yrs ± SD	73 ± 8
Male, *n* (%)	24 (65)
BMI, kg/m^2^ ± SD	28.6 ± 4.3
**Pacing indication**	
Sick sinus syndrome, *n* (%)	4 (11)
AV block, *n* (%)	7 (19)
Pace & ablate, *n* (%)	19 (51)
CRT, *n* (%)	7 (19)
**Comorbidities**	
DMII, *n* (%)	8 (22)
HT, *n* (%)	23 (62)
COPD, *n* (%)	3 (8.)
TIA/CVA, *n* (%)	7 (19)
CAD, *n* (%)	10 (27)
– ACS, *n* (%)	4 (11)
– PCI, *n* (%)	4 (11)
– CABG, *n* (%)	4 (11)
Cardiomyopathy, *n* (%)	22 (60)
– Ischemic, *n* (%)	6 (16)
Atrial fibrillation, *n* (%)	26 (70)
– Paroxysmal, *n* (%)	11 (30)
– Persistent, *n* (%)	12 (32)
– Long‐standing persistent, *n* (%)	3 (8)
**Medication**	
BB, *n* (%)	21 (57)
ACEi, *n* (%)	13 (35)
ARB, *n* (%)	7 (19)
MRA, *n* (%)	7 (19)
Sacubitril/valsartan, *n* (%)	2 (5)
Digoxin, *n* (%)	4 (11)
Dihydropyridin CCB, *n* (%)	7 (19)
Non‐dihydropyridin CCB, *n* (%)	4 (11)
Flecainide, *n* (%)	1 (3)
Sotalol, *n* (%)	3 (8)
Amiodaron, *n* (%)	10 (27)
Acetylsalicylic acid, *n* (%)	4 (11)
Clopidogrel, *n* (%)	2 (5)
VKA, *n* (%)	7 (19)
DOAC, *n* (%)	20 (54)
Diuretics, *n* (%)	11 (30)
**Electrocardiography at implantation**	
Baseline QRS morphology	
– Narrow QRS or RBBB (nQRS), *n* (%)	25 (68)
– LBBB, *n* (%)	12 (32)
QRS duration (ms) ± SD	117 ± 35
– Narrow QRS (ms) ± SD	98 ± 20
– LBBB (ms) ± SD	159 ± 24
Atrial Rhythm	
– SR, *n* (%)	23 (62)
– AF, *n* (%)	11 (30)
– AT, *n* (%)	1 (3)
– Atrial paced, *n* (%)	2 (5)
AV conduction	
– Normal (PR < 200 ms or AF with normal ventricular conduction), *n* (%)	24 (65)
– Prolonged (PR > 200 ms), *n* (%)	10 (27)
– 3rd degree AV block, *n* (%)	1 (3)
– Ventricular paced, *n* (%)	1 (3)
– AF with slow ventricular conduction, *n* (%)	1 (3)
**Echocardiography**	
LVEF (%) ± SD	46 ± 11
– LVEF ≥ 50%, *n* (%)	15 (41)
– LVEF 40‐50%, *n* (%)	13 (35)
– LVEF < 40%, *n* (%)	9 (24)
LVEDD (mm) ± SD	51 ± 6
LVESD (mm) ± SD	38 ± 9
IVS (mm) ± SD	9 ± 2

Abbreviations: ACEi, angiontensin converting enzyme inhibitor; ACS, acute coronary syndrome; AF, atrial fibrillation; Aflut, atrial flutter; AT, atrial tachycardia; ARB, angiotensin recepter blocker; AV, atrioventricular; BB, betablocker; BMI, body mass index; CAD, coronary artery disease; CCB, calcium channel blocker; CABG, coronary artery bypass grafting; COPD, chronic obstructive pulmonary disease; CRT, cardiac resynchronization therapy; CVA, cerebrovascular accident; DMII, diabetes mellitus type II; DOAC, direct oral anticoagulant; HFmrEF, heart failure with mildly reduced ejection fraction; HFrEF, heart failure with reduced ejection fraction; HT, hypertension; IVS, interventricular septum; LBBB, left bundle branch block; LVEF, left ventricular ejection fraction; LVEDD, left ventricular end‐diastolic dimension; LVESD, left ventricular end‐systolic dimension; MRA, mineralocorticoid receptor antagonist; PCI, percutaneous coronary intervention; SR, sinus rhythm; TIA, transient ischemic attack; VKA, vitamin K antagonist.

### 3.2 Procedure‐related characteristics

LBBAP was successful in 33 (89%) patients. The patients with a failed LBBAP procedure had an indication for CRT and underwent successful BiV‐CRT implantation and did not undergo study measurements. In the 33 LBBAP patients, LBB capture was achieved in 21 (63%) patients. Twelve (36%) patients were treated with LVSP. Average paced QRS duration for the entire cohort was 152 ± 18 ms. Extensive procedure‐related characteristics, including capture criteria, are presented in Supplementary data 1.

### 3.3 Electrocardiography

**Figure 3:**
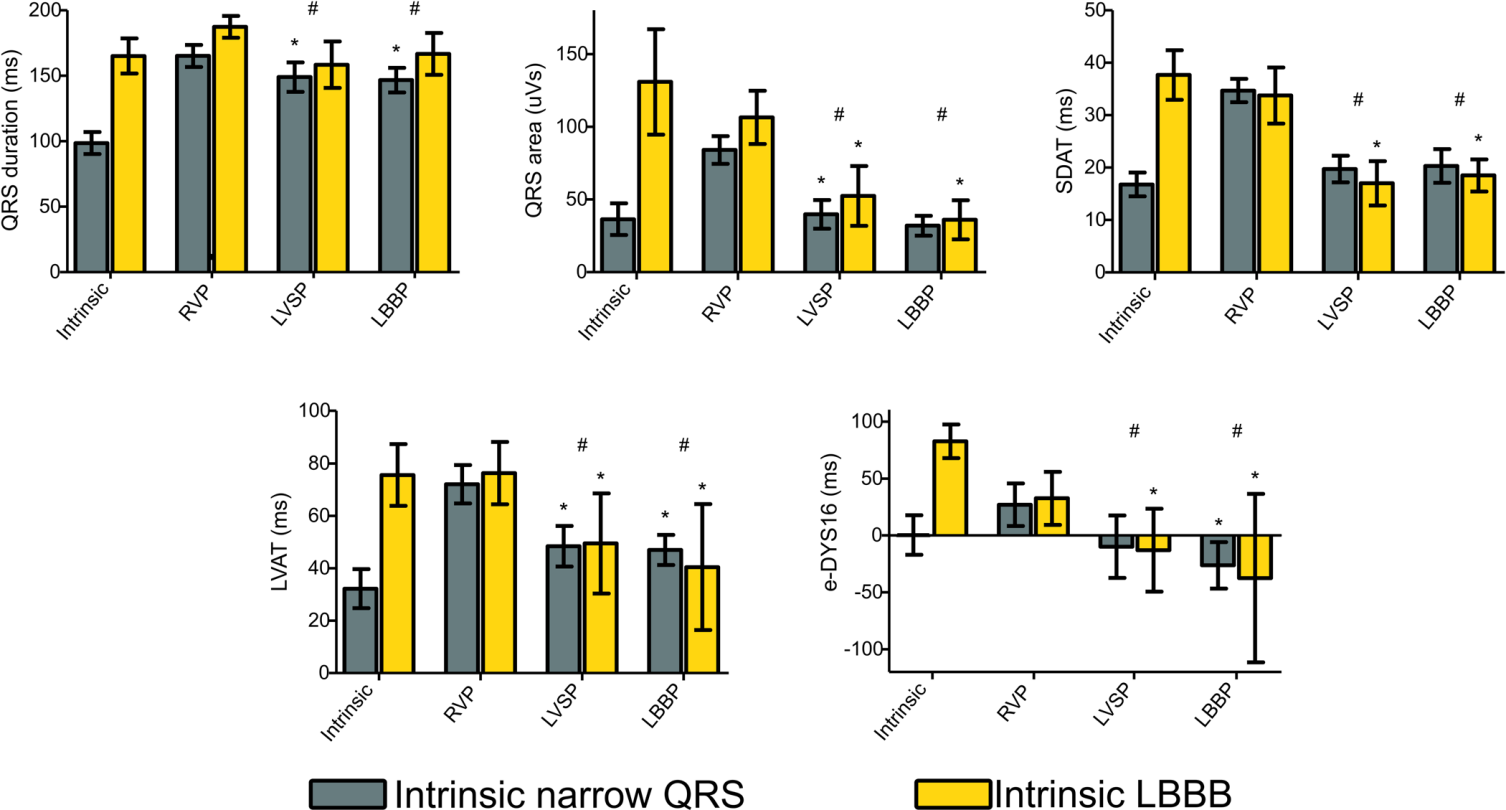
QRS duration, QRS area, SDAT, LVAT & e‐DYS16 for the different pacing sites. SDAT = standard deviation of activation times; LVAT, left ventricular activation time; e‐DYS16, total electrical dyssynchrony between leads V1‐V6; LBBB, left bundle branch block; RVP, right ventricular pacing; LVSP, left ventricular septal pacing; LBBP, left bundle branch pacing. *p < .05 compared to intrinsic; ^#^ *p* < .05 compared to RV pacing (narrow QRS and LBBB pooled).

Both LVSP and LBBP resulted in a significantly longer QRS duration (148 [137, 158] ms and 148 [137, 157] ms, respectively) than baseline narrow QRS (nQRS) (99 [91, 106] ms) (Figure 3 *).* In patients with baseline LBBB, there was no significant difference in QRS duration between intrinsic conduction (159 [146, 171] ms) and both LVSP (159 [145, 172] ms) and LBBP (167 [151, 183] ms) (Figure 3 *).* In the entire cohort, both LVSP and LBBP resulted in a significantly shorter QRS duration (mean difference of 22[11,32] ms and 18[8,28] ms, respectively, *p* < .001 for both) when compared to RVP *(*Figure 3 *).* QRS duration was not significantly different between LVSP and LBBP (*p* = .46) (Figure 3).

### 3.4 Vectorcardiography

During LBBP QRS area was comparable to nQRS (32 [25,40] µVs vs. 29 [23,36] µVs, respectively, *p* = .45), whereas QRS area was significantly larger during LVSP when compared to nQRS (41[32,50] µVs vs. 29[23,36] µVs, respectively, *p* = .018) *(*Figure 3 *)*. In patients with LBBB at baseline (117 [85, 135] µVs), both LVSP (47 [23,72] µVs) and LBBP (47 [20,75] µVs) reduced QRS area significantly (Figure 3 *).* In the entire cohort, QRS area was significantly lower during both LVSP and LBBP when compared to RVP with a mean difference of 52[36,69] µVs and 54[38,70] µVs, respectively (*p* < .001 for both). In the entire cohort, QRS area was not significantly different between LVSP and LBBP (*p* = .81) (Figure 3).

### 3.5 ECG belt

During both LVSP (20[16,24] ms) and LBBP (20[17,23] ms) SDAT was comparable to nQRS (17[15,20] ms) (Figure 3 *).* In patients with LBBB (38 [23,42] ms) at baseline, both LVSP (17 [12,22] ms) and LBBP (19 [14,24] ms) reduced SDAT significantly. In the entire cohort, during both LVSP (19[16,22] ms) and LBBP (20[17,23] ms) SDAT was significantly lower when compared to RVP (35[32,37] ms) (*p* < .001 for both). In the entire cohort, SDAT was not significantly diferent between LVSP and LBBP(*p* = .6) (Figure 3 *).*

In patients with nQRS, LVAT was significantly longer during both LVSP (48[40,57] ms) and LBBP (47[39,54] ms) when compared to nQRS (30[24,36] ms) (*p* < .001 for both) (Figure 3 *).* In patients with LBBB (76 [65,86] ms) at baseline, both LVSP (51 [39,62] ms) and LBBP (43 [30,56] ms) significantly reduced LVAT (Figure 3 *).* In the entire cohort, LVAT was significantly lower during both LVSP (49[41,57] ms) and LBBP (46[37,54] ms) when compared to RV pacing (73[67,79] ms) (*p* < .001 for both) *(*Figure 3 *).* In the entire cohort, LVAT was not significantly different between LVSP and LBBP (*p* = .58) (Figure 3).

### 3.6 UHF‐ECG

During LVSP (−14[−37, 9] ms), e‐DYS16 was comparable to nQRS (0[−15, 15] ms), although a trend towards a negative e‐DYS16 was observed (*p* = .24) (Figure 3 *).* e‐DYS16 was significantly more negative during LBBP (−27[−44, −9] ms) when compared to patients with nQRS (0[−15, 15] ms) (*p* = .008) (Figure 3 *).* In patients with baseline LBBB (81[61, 101] ms), e‐DYS16 was significantly more negative during both LVSP (−13[−35, 9] ms) and LBBP (−34[−64, −5] ms) (*p* < .001 for both) (Figure 3 *).* In the entire cohort, e‐DYS16 was positive during RVP, whereas a negative e‐DYS16 was observed during both LVSP and LBBP (Figure 3 *).* e‐DYS16 was not significantly different between LVSP and LBBP for the entire cohort (*p* = .36).

### 3.7 Unipolar LBBAP versus bipolar LBBAP with anodal stimulation

Bipolar LBBAP with anodal stimulation (aLBBAP) was achieved in 25 patients. aLBBAP was compared to either LVSP or LBBP, depending on the output at which aLBBAP was achieved and the unipolar capture type at this output (LVSP or LBBP). This resulted in comparison with unipolar LBBP in 13 (52%) patients and unipolar LVSP in 12 (48%) patients.

**Figure 4:**
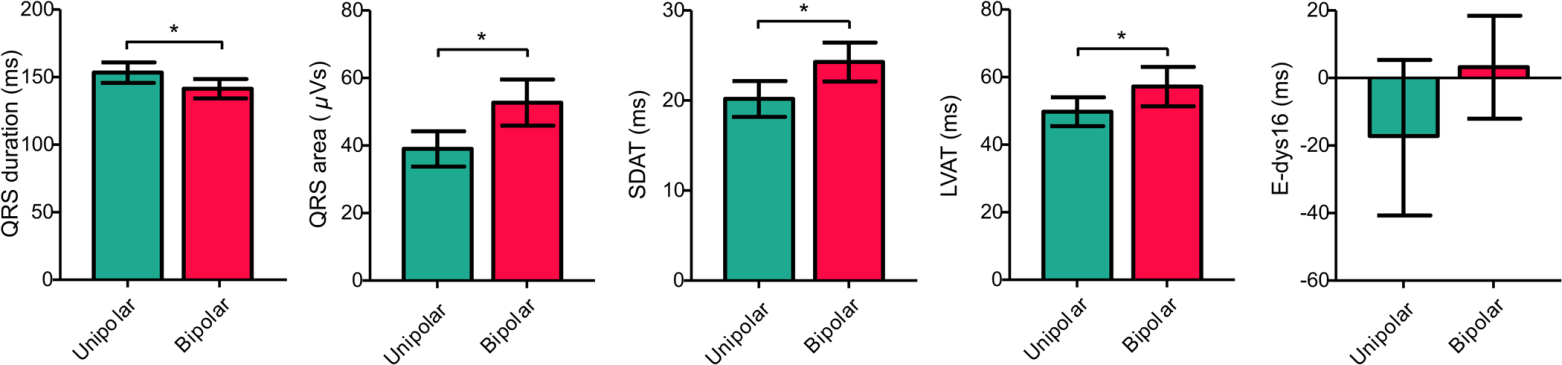
Unipolar LBBAP vs bipolar LBBAP. SDAT, standard deviation of activation times; LVAT, left ventricular activation time; e‐DYS16, total electric dyssynchrony between leads V1–V6. *p < .05.

During aLBBAP, QRS duration was significantly shorter when compared to unipolar LBBAP (141 ± 17 ms vs 153 ± 18 ms, *p* < .001) (Figure 4 *).* QRS area, SDAT and LVAT increased significantly during aLBBAP when compared to unipolar LBBAP (*p* < .001, *p* < .001 and *p* = .029, respectively) (Figure 4 *).* No significant difference was found in terms of e‐DYS16 between aLBBAP and unipolar LBBAP, although a trend towards a less delayed RV was visible (*p* = .075) *(*Figure 4 *)*.

## 4 DISCUSSION

This is the first study exploring the differences in ventricular electrical activation between LBBP and LVSP using a combination of the 12‐lead ECG, vectorcardiogram, ECG belt and UHF‐ECG. We showed that measures of left ventricular dyssynchrony (QRS area and LVAT) during LVSP and LBBP were lower than those during LBBB and RVP. In patients with narrow QRS, both LVSP and LBBP were comparable to intrinsic activation in terms of SDAT. Moreover, QRS area also comparable between LBBP and narrow QRS and only slightly higher in LVSP when compared to narrow QRS. Both LVSP and LBBP reduced and reversed interventricular dyssynchrony (e‐DYS16) created by an LBBB or RV pacing. Interventricular dyssynchrony was comparable to narrow QRS for LVSP, however a trend towards a more delayed RV was visible. e‐DYS16 was significantly more negative in LBBP when compared narrow QRS, indicating a delayed RV activation. There was no significant difference in ventricular electrical heterogeneity measurements between LVSP and LBBP. Finally, LBBAP with anodal stimulation results in a trend towards improved interventricular dyssynchrony based on QRS duration and e‐DYS16 when compared to unipolar LBBAP. This comes at the cost of increased left ventricular dyssynchrony based on QRS area, SDAT and LVAT.

### 4.1 Global electrical activation

The present study shows that QRS duration during LBBAP is comparable to that during LBBB, which is similar to earlier findings using the same measurement of QRS duration (from pacing stimulus to end of QRS) in LBBAP.^3^ LBBB is a known cause of left ventricular dyssynchrony. Whilst LBBAP significantly improved multiple markers of LV dyssynchrony when compared to an LBBB, QRS duration did not improve with LBBAP when compared to an LBBB. This suggests that QRS duration alone does not sufficiently indicates LV dyssynchrony when the QRS duration is prolonged. The fact that markers of interventricular dyssynchrony show a delayed LV activation in LBBB and a delayed RV activation in LBBAP with no significant difference in QRS duration indicate that QRS duration alone best represents global electrical activation with limited additional information on inter‐ or intraventricular dyssynchrony. This is in line with previous studies on cardiac resynchronization which have shown that QRS duration alone is no good marker of dyssynchrony and subsequent predictor for CRT response. Adding LBBB QRS morphology as an additional selection criterion improved CRT response rate in these patients.^25^, ^26^

SDAT is intended as a metric of global electrical dyssynchrony.^21^ In the present study it seems that the LV is better represented than the RV when comparing SDAT values to the other measurements of ventricular electrical heterogeneity used. Previous studies indicated that SDAT can serve as a more accurate predictor of CRT response than QRS duration.^21^, ^27^ SDAT reduction is correlated with improvement in hemodynamics in terms of LV dp/dt max in a CRT population.^21^ Salden et al. showed that LVSP, without LBB capture confirmation, reduced SDAT significantly compared to an LBBB in a CRT population, with values of SDAT comparable to His bundle pacing.^3^ This reduction is also observed in the present study for both LVSP and LBBP, with SDAT values in LVSP and LBBP comparable to intrinsic activation in patients with narrow QRS. Vijayaraman et al. used SDAT to optimize LBBAP in a population with heart failure and an LBBB.^28^ Similar to the present study, they found that both LVSP and LBBP reduced SDAT significantly when compared to baseline LBBB.^28^ Similarly, they found no difference in SDAT between LVSP and LBBP in an LBBB population.^28^

### 4.2 LV dyssynchrony

The clear differences in QRS area between an LBBB and both left ventricular septal and left bundle branch pacing further supports the idea that QRS area is a marker of LV dyssynchrony. Earlier studies showed that QRS area is a predictor for CRT response^19^ and that QRS area reduction by BiV‐CRT relates to clinical outcome.^29^ In an LBBAP population QRS area correlated significantly with V6RWPT,^9^ an LBB capture criterion. The present study shows that LBBP results in QRS area values comparable to normal intrinsic activation. Only a minor, but significant, increase in QRS area is found during LVSP compared to normal intrinsic activation. Moreover, this study shows that both LVSP and LBBP significantly reduce QRS area when compared to a baseline LBBB or RV pacing. QRS area did not differ significantly between LVSP and LBBP. Heckman et al. studied QRS area in relation to LBBAP in a population with anti‐bradycardia pacing and structural normal hearts.^9^ The largest reduction in QRS area compared to RVP was achieved when an r' was present on the ECG in lead V1.^9^ Allthough they found a small but significant reduction in QRS area in LBBP over LVSP, the previous finding suggests that the largest improvement in LV dyssynchrony occurs when pacing the left side of the IVS.^9^ Unlike the present study, Heckman et al. included patients with structural normal hearts only. Since heart failure might lead to distal conduction system disease, this can influence LV activation in LBBP, resulting in a slightly less synchronous LV activation when compared to patients with structural normal hearts.^30^

We studied ECG Belt derived LVAT as an additional marker of LV dyssynchrony. LVAT is reduced significantly by LVSP and LBBP in both LBBB and RV pacing. A small but significant increase in LVAT is observed during LVSP and LBBP when compared to intrinsic activation in patients with narrow QRS. Although a comparable LVAT between normal intrinsic conduction and LBBP was expected, this small difference cannot be explained comprehensively. Hypothetically this might by due to the signal processing by the software. Moreover, it is imaginable that the left thoracic electrodes do not only represent the left ventricular activation, but also take some of the right ventricular activation into account.

### 4.3 Interventricular dyssynchrony

UHF‐ECG was introduced as a novel technology for measuring ventricular dyssynchrony.^22^ e‐DYS16 reflects the direction of ventricular depolarization with positive values seen when RV activation precedes LV activation and negative values when LV activation precedes RV activation.^22^ Curila et al. showed that LVSP preserves interventricular synchrony better than LBBP, while LBBP accelerates left ventricular lateral wall depolarization and therefore preserves LV synchrony better in patients with normal LV ejection fraction.^8^ The same findings were predicted by simulating the ventricular depolarization pattern produced by the different pacing strategies.^31^ The present study corroborates these findings. UHF‐ECG has also been used to analyze the differences between aLBBAP and unipolar LBBAP, with aLBBAP resulting in an improvement in interventricular dyssynchrony.^23^ Additionally, the present study shows that this goes at the cost of an increased LV dyssynchrony when compared to unipolar LBBAP in terms of QRS area, SDAT and LVAT. A possible explanation for this increase in LV dyssynchrony is that aLBBAP creates ‘pre‐excitation’ of the IVS to some extent. The simultaneous activation of the left and right side of the IVS might result in an earlier activation of a bigger part if the IVS, resulting in an increase in LV dyssynchrony.

### 4.4 Combining measurements of dyssynchrony

The parameters for electrical activation used in the present study have multiple strong and weak points complementing each other. QRS duration can be used as a measurement of global electrical activation, however it is not indicative of inter‐ or intraventricular dyssynchrony. ECG belt derived SDAT is a measurement of global electrical dyssynchrony.^21^ The fact that it can be used as reliable predictor for CRT response, shows that it also takes LV dyssynchrony into account.^21^, ^27^ QRS area can be used as an excellent marker for LV (dys)synchrony, but mostly discards the right ventricle and interventricular dyssynchrony. e‐DYS16 on the other hand is a marker of interventricular (dys)synchrony. Hence, these measurements are complementary to each other when trying to understand the entire electrical activation patterns in different pacing strategies. Combining these parameters enabled us to analyze the differences between intrinsic activation, RV pacing, LVSP, LBBP and aLBBP.

## 5 LIMITATIONS

The results of this study should be interpreted in light of its limitations. This is a small observational study with a heterogeneous cohort of patients. Not all capture types (LVSP, LBBP and aLBBAP) were possible in all patients, making direct comparisons only possible with estimated marginal means created with a linear mixed effects model. The EP system used for ECG recordings for UHF‐ECG had a frequency band of 0.5‐500 Hz. This is lower than the usual band up to 1000 Hz for complete UHF‐ECG analyses. Moreover, the EP system was only compatible with six precordial leads (V1‐V6) instead of the usual eight precordial leads (V1‐V8) for UHF‐ECG analyses. Therefore, e‐DYS16 was used instead of e‐DYS18. Last, recordings for UHF‐ECG were only 1 min (with an average amount of heart beats ~75), were usually >200 beats are used for analyses.

## 6 CONCLUSION

Using a combination of QRS duration, QRS area, SDAT, LVAT and e‐DYS16, LVSP and LBBP result in comparable ventricular electrical heterogeneity. All ventricular dyssynchrony measurements consistently show that both LVSP and LBBP reduce dyssynchrony in patients with LBBB and restore dyssynchrony as a result of RV pacing. Although no significant differences were observed between LVSP and LBBP, when compared to normal intrinsic conduction LVSP slightly increases LV dyssynchrony, while LBBP introduces a marginal increase in interventricular dyssynchrony. Anodal LBBAP reduces interventricular dyssynchrony but increases LV dyssynchrony compared to unipolar LBBAP. QRS duration primarily reflects global electrical activation, while SDAT incorporates LV dyssynchrony. QRS area and LVAT are best suited for assessment of LV (dys)synchrony, while e‐DYS16 is better for evaluating interventricular (dys)synchrony.

## CONFLICT OF INTEREST STATEMENT

F.W. Prinzen reports research grants from Medtronic, Abbott, MicroPort CRM and Biotronik. K. Vernooy reports research and educational grants and consultancy agreements with Medtronic, Abbott, Boston scientific, Microport, Philips and Biosense webster (all grants are paid to the institute). J.G.L.M. Luermans reports a research grant and consultancy agreement with Medtronic (all grants are paid to the institute). K. Curila is co‐founder of the commercial version of the UHF‐ECG system (VDI technologies), filed an US patent No: US 11,517,243B2: “Method of electrocardiographic signal processing and apparatus for performing the method.” and is shareholder of the company VDI Technologies, Inc. R. Cornelussen and S. Gosh are Medtronic employees. S. Gosh is shareholder of Medtronic. J. Lumens reports research grants from Medtronic.

### 1 INSTITUTIONAL REVIEW BOARD STATEMENTS

The study was conducted in accordance with the Declaration of Helsinki, and was approved by the local ethics committee (METC 20‐099), the Dutch Central Committee on Research Involving Human Subjects (CCMO) and the institutional review boards of both participating centers (MUMC and Radboudumc).

### 2 INFORMED CONSENT STATEMENT

Informed consent was obtained from all subjects involved in the study.

## Supporting information

Supporting information.

## Data Availability

The data that support the findings of this study are available from the corresponding author upon reasonable request.
