# Supplementary material for: Assessment of ventricular electrical heterogeneity in left bundle branch pacing and left ventricular septal pacing by using various electrophysiological methods
Source: J Cardiovasc Electrophysiol. 2024 Sep 23;35(12):2282–92. doi: 10.1111/jce.16435 (PMC11650391; doi:10.1111/jce.16435)
Supplement: Supplementary file 1 — Supporting information. [file JCE-35-2282-s001.docx]

**Supplementary data**

|  | Overall (N=37) |
| --- | --- |
| Implant success, n (%) | 33 (89) |
| **Capture** | |
| LVSP only, n (%) | 12 (32) |
| LVSP and LBBP*, n (%) | 5 (14) |
| LBBP only^^^, n (%) | 16 (43) |
| **Location of LBB capture** | |
| - LBBP, n (%) - LPFP, n (%) - LSFP, n (%) - LAFP, n (%) - Unknown, (no LBB potential), n (%) | 4 (11)  5 (14)  3 (8)  0 (0)  9 (24) |
| **LBB capture confirmation** | |
| QRS morphology transition, n (%) | 17 (46) |
| V6 RWPT <75ms (nQRS) or <80ms (LBBB/IVCD), n (%) | 14 (38) |
| V6-V1 interpeak, n (%) | 16 (43) |
| LBBpot-V6RWPT = stim-V6RWPT, n (%) | 12 32) |
|  | |
| LBBpot – QRS (ms) ±SD | 25 ± 12 |
| Paced QRS duration (ms) ± SD | 152 ± 18 |

**Supplementary table 1.** Implantation characteristics. LBBB = left bundle branch block; LVSP = left ventricular septal pacing; LBBP = left bundle branch pacing; LPFP = left posterior fascicle pacing; LSFP = left septal fascicle pacing; LAFP = left anterior fascicle pacing; LBB = left bundle branch; V6 RWPT = time to peak R wave in lead V6; nQRS = narrow QRS; IVCD = interventricular conduction delay; LBBpot = left bundle branch potential. * These are patients in which a capture transition was noticed from non-selective LBBP to LVSP during decremental output pacing. ^ These are the patient with either non-selective LBBP only or patients in which a capture transition was noticed from non-selective LBBP to selective LBBP during decremental output pacing.

| **Baseline narrow QRS (+RBBB)** | | | | |
| --- | --- | --- | --- | --- |
|  | *Intrinsic* | *RVP* | *LVSP* | *LBBP* |
| *QRS duration (ms) [95%CI]* | 99[91,106] | 165[157,163] | 148[137,158] | 148[137,157] |
| *QRS area (µVs) [95%CI]* | 29[23,36] | 68[61,75] | 41[32,50] | 32[25,40] |
| *SDAT (ms) [95%CI]* | 17[15,20] | 35[32,37] | 20[16,24] | 20[17,23] |
| *LVAT (ms) [95%CI]* | 30[24,36] | 72[66,78] | 48[40,57] | 47[39,54] |
| *e-DYS16 (ms) [95%CI]* | 0[-15,15] | 36[20,52] | -14[-37,9] | -27 [-44,-9] |
| **Baseline LBBB** | | | | |
|  | *Intrinsic* | *RVP* | *LVSP* | *LBBP* |
| *QRS duration (ms) [95%CI]* | 159[147,171] | 187[176,199] | 159[145,172] | 167[151,183] |
| *QRS area (µVs) [95%CI]* | 117[85,135] | 93[69,116] | 46[23,72] | 47[20,75] |
| *SDAT (ms) [95%CI]* | 38[23,42] | 34[30,38] | 17[12,22] | 19[14,24] |
| *LVAT (ms) [95%CI]* | 76[23,42] | 77[66,88] | 51[39,62] | 43[30,56] |
| *e-DYS16 (ms) [95%CI]* | 81[61,101] | 48[26,70] | -13[-35,9] | -34[-64,-5] |

**Supplementary table 2.** Ventricular electrical heterogeneity between different pacing strategies using different dyssynchrony measurements, presented as estimated marginal means [95% CI]. RBBB = right bundle branch block; RVP = right ventricular pacing; LVSP = left ventricular septal pacing; LBBP = left bundel branch pacing; SDAT = standard deviation of activation time; LVAT = left ventricular activation time; e-DYS16 = total electrical dyssynchrony between leads V1-V6; LBBB = left bundle branch block.
